# Therapeutic evaluation of low‐dose IL‐2‐based immunomodulatory approach in patients with early AD

**DOI:** 10.1002/alz.092407

**Published:** 2025-01-09

**Authors:** Marie Sarazin, Julien Lagarde, Pauline Olivieri, Virginie Puchois, Laetitia Federici, Thomas Chaigneau, Marie‐Claude Potier, Sylvain Auvity, Michel Bottlaender, Guillaume Dorothée

**Affiliations:** ^1^ Neurology of Memory and Language Department, GHU Paris Psychiatrie & Neurosciences, Hôpital Sainte‐Anne, Paris France; ^2^ Université Paris‐Saclay, CEA, CNRS, Inserm, BioMaps, Orsay France; ^3^ Université Paris Cité, Paris France; ^4^ Inserm, Sorbonne Université, Centre de Recherche Saint‐Antoine, Immune System and Neuroinflammation Laboratory, Hôpital Saint‐Antoine, Paris France; ^5^ Université Paris Cité, Inserm, Therapeutic Optimization in Neuropsychopharmacology, Paris France; ^6^ Department of Pharmacy, AP‐HP, Hôpital Necker, Paris France; ^7^ Paris Brain Institute, ICM, Cnrs, Inserm, Sorbonne Université, Hôpital de la Pitié‐Salpêtrière, Paris France; ^8^ Université Paris‐Saclay, UNIACT, Neurospin, Joliot Institute, CEA, Gif‐sur‐Yvette France

## Abstract

**Background:**

Chronic innate neuroinflammation mediated by microglia and astrocytes in response to Aβ and pathological Tau species is a cardinal feature of AD that contributes to disease pathogenesis. Accumulating evidence now also highlight an instrumental role of T cells and peripheral‐central immune crosstalk in the pathophysiology of AD. Both preclinical and clinical reports suggest the potential therapeutic interest of peripheral immunomodulatory approaches aimed at amplifying regulatory T cells (Tregs), e.g. through low‐dose IL‐2 treatment, as a strategy for rebalancing T cell subsets and proper central innate neuroinflammation towards beneficial neuroinflammatory patterns.

**Method:**

Our study aims at evaluating the therapeutic efficacy and safety of low‐dose IL‐2 immunomodulatory treatment in 35 patients with early AD, in a randomized (2:1), double blind, placebo‐controlled phase II clinical trial. Treatment consists in 21 cures of subcutaneous injections of either placebo or low‐dose of IL‐2 (PROLEUKIN®) at 1 MIU/day for 5 consecutive days for the first week (induction phase), then one injection per week for 16 weeks from day 15 (maintenance phase; total duration of treatment: 18 weeks). Patients are followed‐up for cognitive and functional assessment at 6, 12 and 18 months after the first injection. All subjects undergo [^18^F]‐DPA‐714‐PET/MRI at inclusion and 18 months after treatment induction, for monitoring the evolution of brain neuroinflammation.

**Result:**

An intermediate analysis of the variation of Tregs percentage was performed after 9 patients were included and treated for 4 doses into the maintenance phase. Percentages of Tregs, among both CD4+ or total CD3+ T cells, showed a 1.5‐fold increase in the treated group (n = 6), while remaining stable in the placebo group (n = 3), as expected. One patient decided to discontinue the treatment because of depression, which appears unlikely to be treatment‐related. No other adverse effect was observed.

**Conclusion:**

Our intermediate biological analysis evidence a 1.5‐fold increase in Tregs percentages after 4 doses of the maintenance phase, without any significant adverse effect. These data validate our predefined objective of Treg amplification and thus target engagement, while supporting optimal safety of the treatment.

